# Overoxidation and Oligomerization of *Trypanosoma cruzi* Cytosolic and Mitochondrial Peroxiredoxins

**DOI:** 10.3390/pathogens12101273

**Published:** 2023-10-23

**Authors:** María Dolores Piñeyro, María Laura Chiribao, Diego G. Arias, Carlos Robello, Adriana Parodi-Talice

**Affiliations:** 1 Laboratorio de Interacciones Hospedero Patógeno, Unidad de Biología Molecular, Institut Pasteur de Montevideo, Montevideo 11400, Uruguay; mpineyro@fmed.edu.uy (M.D.P.); chiribao@fmed.edu.uy (M.L.C.); robello@pasteur.edu.uy (C.R.); 2 Departamento de Bioquímica, Facultad de Medicina, Universidad de la República, Montevideo 11800, Uruguay; 3 Laboratorio de Enzimología Molecular, Instituto de Agrobiotecnología del Litoral, UNL-CONICET, Santa Fe 3000, Argentina; darias@fbcb.unl.edu.ar; 4 Facultad de Bioquímica y Ciencias Biológicas, Universidad Nacional del Litoral, Santa Fe 3000, Argentina; 5 Sección Genética Evolutiva, Instituto de Biología, Facultad de Ciencias, Universidad de la República, Iguá 4225, Montevideo 11400, Uruguay

**Keywords:** *Trypanosoma cruzi*, peroxiredoxin, overoxidation, chaperone activity, oligomerization

## Abstract

Peroxiredoxins (Prxs) have been shown to be important enzymes for trypanosomatids, counteracting oxidative stress and promoting cell infection and intracellular survival. In this work, we investigate the in vitro sensitivity to overoxidation and the overoxidation dynamics of *Trypanosoma cruzi* Prxs in parasites in culture and in the infection context. We showed that recombinant m-TXNPx, in contrast to what was observed for c-TXNPx, exists as low molecular mass forms in the overoxidized state. We observed that *T. cruzi* Prxs were overoxidized in epimastigotes treated with oxidants, and a significant proportion of the overoxidized forms were still present at least 24 h after treatment suggesting that these forms are not actively reversed. In in vitro infection experiments, we observed that Prxs are overoxidized in amastigotes residing in infected macrophages, demonstrating that inactivation of at least part of the Prxs by overoxidation occurs in a physiological context. We have shown that m-TXNPx has a redox-state-dependent chaperone activity. This function may be related to the increased thermotolerance observed in m-TXNPx-overexpressing parasites. This study suggests that despite the similarity between protozoan and mammalian Prxs, *T. cruzi* Prxs have different oligomerization dynamics and sensitivities to overoxidation, which may have implications for their function in the parasite life cycle and infection process.

## 1. Introduction

*Trypanosoma cruzi* causes Chagas disease that affects 6–7 million people worldwide [1]. It is a life-threatening disease that is a major concern in endemic areas and an emerging threat to public health in non-endemic countries. Currently used drugs to treat Chagas disease cause severe side-effects, are outdated, and effective only in the acute phase of the disease [2]. During their life cycle, parasites such as *T. cruzi* cope with numerous environmental changes and different types of physical and chemical stress. One important stress they face is oxidative stress, both in the insect vector’s gut and in the mammalian host. The parasites express a number of antioxidant proteins that are part of trypanosomatid-specific systems and are based on a low molecular mass dithiol, trypanothione. The cytosolic and mitochondrial tryparedoxin peroxidases of *T. cruzi* (c-TXNPx and m-TXNPx, respectively) are 2 cysteine (2-cys) peroxiredoxins, that are part of this system and are capable of detoxifying hydrogen peroxide and peroxynitrite generated in the compartments in which they are expressed [3].

Peroxiredoxins (Prxs) have been shown to be key enzymes for trypanosomatids. Tryparedoxin peroxidase from *Trypanosoma brucei* is essential for parasite viability and proliferation in culture [4]. RNA interference experiments in *T. brucei* have shown that the cytosolic isoform is essential for the blood form of the parasite [4]. *T. cruzi* parasites overexpressing cytosolic and mitochondrial Prxs were able to replicate more efficiently in phagocytic and nonphagocytic cells in vitro, suggesting that Prx activities are associated with parasite infectivity [5]. Both *Leishmania donovani* and *T. cruzi* c-TXNPx-overexpressing lines were associated with increased resistance to exogenous H_2_O_2_ and peroxynitrite [6,7].

In addition, several drugs currently used in chemotherapy for Chagas disease induce changes in the redox balance of these parasites [8]. Consequently, the systems involved in maintaining this balance, including Prxs, are important for ensuring survival and successful infection.

Recently, we showed that recombinant c-TXNPx exists in different oligomeric forms, from decameric to high molecular mass (HMM) aggregates, and functions as a dual-function protein, acting as both a peroxidase and a molecular chaperone [9]. Chaperone activity has also been demonstrated for mitochondrial Prxs from *Leishmania infantum* and *T. cruzi* [10,11]. In *L. infantum*, this activity may be relevant for infection and survival in vivo, as knock-out parasites for m-TXNPx were reported to be more sensitive to temperature changes and avirulent in a murine infection model [11]. Interestingly, virulence was restored in a strain expressing Prx without peroxidase activity [11].

It has been reported that overoxidation of sulfenic (-SOH) acid to sulfinic (-SO_2_H) or sulfonic (-SO_3_H) acid of the peroxidatic cysteine of the 2-Cys Prx causes inactivation of the enzyme [12]. Previously, we have shown that recombinant c-TXNPx protein can be overoxidized in the presence of H_2_O_2_, which affects its quaternary structure and promotes the formation of HMM forms [9]. The relevance of oligomerization and overoxidation dynamics in the replication and infection processes of the parasite remains unknown.

Here, we aimed to compare the oligomerization dynamics of c-TXNPx with that of m-TXNPx in relation to redox state, susceptibility to overoxidation, and their role as peroxidase and/or chaperone. We studied the sensitivity of both tryparedoxin peroxidases to oxidants and the dynamics of overoxidation of these enzymes in the epimastigote forms of *T. cruzi* as well as during the infection process, using in vitro cell cultures.

## 2. Materials and Methods

### 2.1. Parasite Culture and Genetic Manipulation

Epimastigotes of the *Dm*28c strain were grown in liver infusion tryptose medium (LIT) supplemented with 10% heat-inactivated fetal bovine serum at 28 °C. Trypomastigotes were harvested from the supernatant of monolayers of infected Vero cells. Amplifications of the Prxs genes were performed as previously described [3] and cloned in the pGEM-T vector. The entire gene was excised with *Eco*RI and *Hind*III restriction enzymes and ligated into the trypanosomal expression vector pTREX, resulting in pTREX–c-TXNPx and pTREX-m-TXNPx plasmids [13]. For transformation, 7 × 10^7^ epimastigotes were resuspended in HBS buffer (21 mM HEPES, 137 mM NaCl, 5 mM KCl, 7 mM Na_2_HPO_4_, and 6 mM glucose at pH 7.4) and transfected with 100 μg of each pTREX plasmid (including empty pTREX as a control) by electroporation in a BTX Electro Cell Manipulator 600 using two pulses at 450 V, 1300 μF, and 13 Ω in 4 mm cuvettes. The transfected parasites were grown in the presence of continuously increasing G418 (GIBCO) to a final concentration of 500 μg/mL. The cultures were maintained in LIT at 200 μg/mL of G418. The overexpression of Prxs was confirmed by Western blot.

### 2.2. Gel Filtration Chromatography

Gel filtration chromatography was performed at 25 °C using a HPLC chromatograph on a TSK-GEL G4000SWXL column (Supelco, USA). Thyroglobulin (669 kDa), ferritin (440 kDa), aldolase (158 kDa), and conalbumin (75 kDa) were used as molecular mass standards (GE Healthcare Calibration Kit). The column was equilibrated with 50 mM sodium phosphate (pH 7.4) and 100 mM NaCl at a flow rate of 1 mL/min. The purified proteins were diluted in the range of 1–3 mg/mL. The sample volume for each run was 50 μL. The absorbance at 280 nm was measured to monitor protein elution profiles. Protein-containing fractions were recovered for further analysis.

### 2.3. Expression, Purification, and Treatment of Recombinant Proteins

Wild-type cytosolic and mitochondrial tryparedoxin peroxidases were expressed and purified, as previously described [9]. The proteins were incubated in the presence of 10 mM DTT (reduction condition) or in the presence of H_2_O_2_ at a 1:1 molar ratio (oxidation condition). Prx overoxidation was performed by inactivating the protein by multi-turnover cycling, with 10 mM H_2_O_2_ and 10 mM DTT.

### 2.4. Peroxidase Activity Assay

Peroxidase activity was measured by monitoring NADPH oxidation at 340 nm using a coupled assay to regenerate Prxs in their reduced form. The overoxidation index (*f inact*), which measures the fraction of peroxiredoxin inactivated during each catalytic cycle, was calculated as previously described [14]. The *f inact* data were used to calculate C_hyp1%_ which corresponds to the peroxide concentration at which 1 out of every 100 TXNPx molecules is inactivated by overoxidation per turnover, as previously described [14].

### 2.5. Molecular Chaperone Activity Assay

Molecular chaperone activity was determined by assessing the ability of recombinant m-TXNPx to inhibit the thermal aggregation of malate dehydrogenase (MDH) (Sigma-Aldrich, USA). Briefly, m-TXNPx (1:10 molar ratio of MDH:m-TXNPx) with different treatments (reduction, oxidation, and overoxidation) was incubated in 50 mM HEPES (pH 7.6) containing 10 mM NaCl, at 45 °C, for 5 min with agitation, to ensure stability of the protein solution. After 5 min, 1 μM MDH was added to the solution and the reaction was incubated for 20 min. The effect of bovine serum albumin (BSA) (10 µM) on the MDH aggregation was used as a negative control. The thermal aggregation of MDH was monitored by light scattering at 340 nm using a Cary Eclipse Fluorescence Spectrophotometer (Varian Inc., USA) for 10 min.

### 2.6. Gel Electrophoresis and Western Blot

Protein extracts were obtained from parasite cultures incubated in the absence or presence of H_2_O_2_. These extracts were resolved in gels, transferred to nitrocellulose membranes, and incubated with various primary antibodies. The antibodies used were anti-PrxSO_2/3_ (AbFrontiers, USA or Cambridge Research Biochemicals, dilution 1/1000 or 1/2000) and a rabbit sera anti-c-TXNPx (dilution 1/5000) or anti m-TXNPx (1/1000). After washes, membranes were incubated with HRP-conjugated anti rabbit (dilution 1/30,000) as the secondary antibody. Chemiluminescence was developed with SuperSignal West Pico Plus Chemiluminescent Substrate (Thermo Scientific, USA) and detected with ImageQuant 800. Native-PAGE was performed using a NativePAGE™ Novex^®^ Bis-Tris Gel System (Life Technology, USA) following the manufacturer’s instructions.

### 2.7. Indirect Immunofluorescence Microscopy

Epimastigotes were resuspended in glucose 1% (*w*/*v*) in phosphate-buffered saline (PBS) at 1 × 10^7^ parasites/mL and treated with 500 µM *tert*-butyl hydroperoxide (*t*-BOOH) for 30 min. After treatment, parasites were washed with PBS and fixed with 4% paraformaldehyde in PBS for indirect immunofluorescence assay (IIF). IIF was performed using a mouse sera anti-c-TXNPx (1/200) or mouse sera anti-mTXNPx (1/200) in combination with rabbit anti-PrxSO_2/3_ (1/1000) antibody (ABFrontiers, USA). Confocal images were analyzed with Fiji (https://imagej.net/Fiji, accessed on 22 August 2023) [15]. Colocalization analysis was conducted using the Coloc2 plugin in ImageJ, yielding Pearson’s R value and Manders’ tM (above threshold) coefficients from regions of interest. Pearson’s R values, tM1, and tM2 are reported as mean ± standard deviation values from 10 regions of interest. Colocalization colormaps were obtained with the colocalization colormap plugin.

THP1 monocytes were cultured in RPMI supplemented with 10% heat-inactivated fetal bovine serum (FBS) and 2 mM 2-mercaptoethanol at 37 °C under 5% CO_2_. For macrophage differentiation, 60,000 monocytes per well were seeded onto coverslips in 12-well plates in complete RPMI medium with 25 nM Phorbol 12-myristate 13-acetate (PMA) for 48 h. After differentiation, macrophages were infected with 300,000 *Dm*28c *T. cruzi* trypomastigotes per well for 2 h and washed 3 times with PBS. Cells were fixed with 4% paraformaldehyde in PBS for 1 h at room temperature (RT), washed twice with PBS, and permeabilized with Triton-X 100 10% (*v*/*v*) at RT for 10 min. The cells were then incubated with 50 mM NH_4_Cl for 10 min at RT, washed twice with PBS, and blocked with 2% bovine serum albumin (BSA) at RT for 1 h. After blocking, the coverslips were incubated with mouse sera anti-cTXNPx (dilution 1/500), anti-mTXNPx (dilution 1/300), and rabbit anti-PrxSO_2/3_ (dilution 1/1000) antibodies in a blocking solution for 1 h at RT. After three washes with PBS-0.1% Tween 20, the coverslips were incubated with Alexa-488 conjugated anti-rabbit antibody and Cy3 conjugated anti-mouse antibody (both diluted 1/800 in PBS 1% BSA 0.1% Tween20) for 1 h at RT. After washing, the coverslips were mounted with Fluoroshield containing DAPI (Sigma-Aldrich, USA) and visualized using a Leica TCSSP5 confocal microscope.

### 2.8. Detection of Overoxidized TXNPx in Parasites

To detect overoxidized TXNPx in parasites, epimastigotes in the exponential phase were centrifuged, washed three times with PBS, incubated in PBS glucose 1% (*w*/*v*), and treated with H_2_O_2_ at different concentrations (0, 100, 200, and 500 µM) for 30 min at 28 °C. The treated parasites were lysed at different times. The procedure was as follows: time 0 corresponded to parasites which, after incubation for 30 min a 28 °C, were centrifuged, suspended in Hepes-NaOH 50 mM pH 7.4, 1 mM EDTA, 1 mM DTT, and proteases inhibitors, and lysed; recovery time 4 h corresponded to parasites that, after incubation with H_2_O_2,_ were centrifuged and suspended in LIT medium supplemented with FCS 10% and incubated for 4 h at 28 °C. Recovery time 24 h corresponded to parasites recovered, after H_2_O_2_ treatment, and incubated in LIT medium for 24 h at 28 °C. The treated parasites were processed in identical form as at time 0.

Experiments were performed with the same time scheme as above with the following modifications: the treated parasites were centrifuged and resuspended in Hepes 40 mM pH 7.4, 1 mM EDTA, N-ethylmaleimide (NEM), 200 U/mL catalase (Sigma, USA), and protease inhibitors for 10 min. After centrifugation, the parasites were lysed in Tris pH 8.0, 150 mM NaCl, and NP40 0.1% (*w*/*v*).

Native protein extracts were prepared from the treated parasites. Briefly, the parasites were disrupted by three cycles of thawing/frosting in liquid nitrogen. Cell debris was removed by centrifugation at 15,000× *g* for 15 min. The native extracts were dialyzed against Hepes 50 mM pH 7.4, 1 mM EDTA to remove DTT. The crude protein extracts were subjected to native-PAGE or SDS-PAGE and their overoxidation state and structural properties were analyzed by immunoblotting as described in the Western blot section.

To detect overoxidation in cell-derived trypomastigotes parasites, the obtained trypomastigotes (30 × 10^6^) were centrifuged and resuspended in PBS in the absence or presence of different concentrations of *t*-BOOH during 30 min at RT. The parasites were lysed in the same manner as the epimastigotes, and extracts were resolved by SDS-PAGE. The parasite protein extracts were quantified using the Bradford method.

### 2.9. Thermotolerance Assay

Epimastigote cultures in the exponential phase of growth were centrifuged at 2000× *g* for 10 min, resuspended at 3 × 10^6^ cells/mL in LIT medium plus SBF 10% (*v*/*v*), and incubated for 72 h at 28 °C or 37 °C. The growth values at 37 °C were normalized to 28 °C after measuring cell growth using the resazurin method as described by Rolón et al. [16].

### 2.10. In Vitro Infection Assay

Semi-confluent Vero cells (70%) were infected with cell-derived trypomastigotes from wild-type or TXNPx-overexpressing lines at a ratio of five parasites per cell in 12-well plates with coverslips. The cells were infected in DMEM supplemented with FBS 10% (*v*/*v*). After infection, the cells were washed twice with PBS and the medium was replaced with fresh medium. The coverslips were removed at 48 h post infection and fixed with PFA 4% (*v*/*v*) for 20 min at RT. The cells were mounted with Fluoroshield containing DAPI (Sigma) and the number of infected cells and amastigotes per infected cell was determined by counting the nuclei of Vero cells and the kinetoplast of amastigotes using epifluorescence microscopy. The analysis was performed with Icy software [17]. For some experiments, trypomastigotes from the different recombinant lines were previously incubated with different concentrations of H_2_O_2_ or *t*-BOOH for 30 min and then were used for infection experiments.

### 2.11. Statistical Analysis

Statistical analysis was performed using Student’s *t*-test for unpaired data. Results were considered significant when *p* < 0.05. Data are expressed as means ± SEM.

## 3. Results

### 3.1. Recombinant c-TXNPx and m-TXNPx Present Differences in Oligomerization under Oxidative Conditions

**Figure 1:**
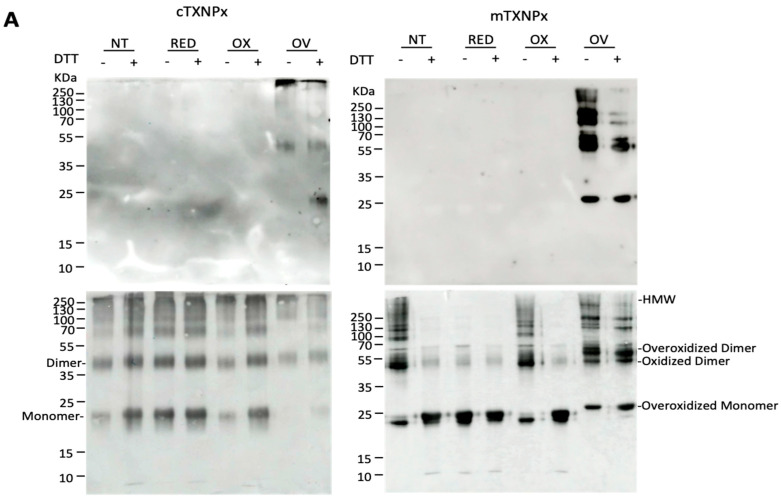

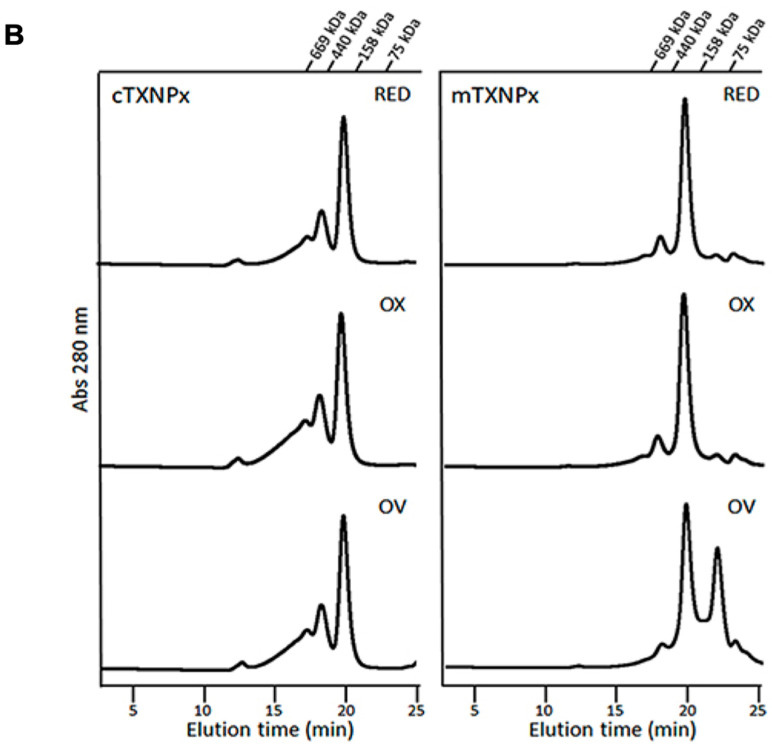
(**A**) Analysis of recombinant c-TXNPx and m-TXNPx under different redox conditions by immunoblotting. Proteins were treated under the following conditions: reduction (RED): 10 mM DTT; oxidation (OX): H_2_O_2_ (1:1 molar ratio); overoxidation (OV): 10 mM DTT and 10 mM H_2_O_2_; NT: proteins without treatment. Proteins were resolved by SDS-PAGE, transferred to nitrocellulose membranes, and incubated with anti-Prx-SO_3_ (upper images) and anti-TXNPx antibodies (lower images). (**B**) Gel filtration chromatograms of the recombinant proteins c-TXNPx and m-TXNPx. A total of 150 μg of protein was applied to a TSK-GEL G4000SW_XL_ column (Supelco). Elution profiles of RED: reduced Prxs; OX: oxidized Prxs; OV: overoxidized Prxs. Elution times of standard proteins were as follows: thyroglobulin (669 kDa) 16.80 min; ferritin (440 kDa) 19.02 min; aldolase (158 kDa) 21.12 min; and ovalbumin (75 kDa).

Previously, we showed that recombinant c-TXNPx protein is overoxidized and oligomerized to HMM forms after H_2_O_2_ treatment [9]. Here, we aimed to compare the oligomerization dynamics of cytosolic and mitochondrial Prxs. Recombinant proteins were subjected to different redox conditions: reduction, oxidation, overoxidation, or no treatment, and then subjected to SDS-PAGE and immunoblotting using anti-PrxSO_2/3_ and anti-c-TXNPx and mTXNPx antibodies. For all conditions except overoxidation, the specific antibody against c-TXNPx detected the protein as a monomer, dimer, and HMM oligomers, regardless of whether DTT was added prior to loading the samples onto the gel, suggesting that oligomers are stabilized for other interactions rather than disulfide bonds, which appear to be strong enough not to break under denaturing conditions. In contrast, m-TXNPx showed several oligomers that became monomers upon reduction, indicating that dimers and oligomers are stabilized by disulfide bridges. For c-TXNPx, a large proportion of the overoxidized forms corresponded to high-mass oligomers, whereas for m-TXNPx, oligomers of lower molecular mass (LMM), even monomers, were observed (Figure 1A and Supplementary Figure S1). Remarkably, the migration patterns of m-TXNPx monomers and dimers varied depending on the treatment, with the overoxidized monomers and dimers exhibiting a lower mobility compared to the other conditions. These dimers and monomers appear to represent intermediate species with at least one overoxidized peroxidatic cysteine, preventing disulfide bond formation, and are expected to adopt a more extended structure that migrates with lower mobility on SDS-PAGE. As for m-TXNPx in the oxidation condition, without DTT, the majority corresponded to oligomers of LMM up to the decamer, including an oxidized dimer with two disulfide bonds, which migrated more than in the overoxidized condition. In the overoxidized state, the majority of oligomers are overoxidized and, therefore, do not change in the presence of a reducing agent. Under this condition, the monomers and dimers of m-TXNPx migrate less during electrophoresis and cannot form disulfide bonds. Gel filtration of both recombinant proteins was performed to obtain the oligomerization profile. In agreement with SDS-PAGE experiments, overoxidation conditions produced both decamers and HMM forms for c-TXNPx, whereas in the case of m-TXNPx, mainly decamers, and a small proportion of oligomers of lower mass compatible with a tetramer or dimer were present (Figure 1B). In the reduced and oxidized states, m-TXNPx eluted mainly as a peak with the molecular mass of a decamer and a minor peak of a HMM. Eluted peaks, analyzed using native-PAGE and Western blot, confirmed this oligomeric behavior. These results suggest that although both enzymes have similar quaternary structures, they exhibit different oligomerization dynamics under oxidative stress.

### 3.2. T. cruzi m-TXNPx Exhibits Molecular Chaperone Activity Dependent on Its Oxidation Status

**Figure 2:**
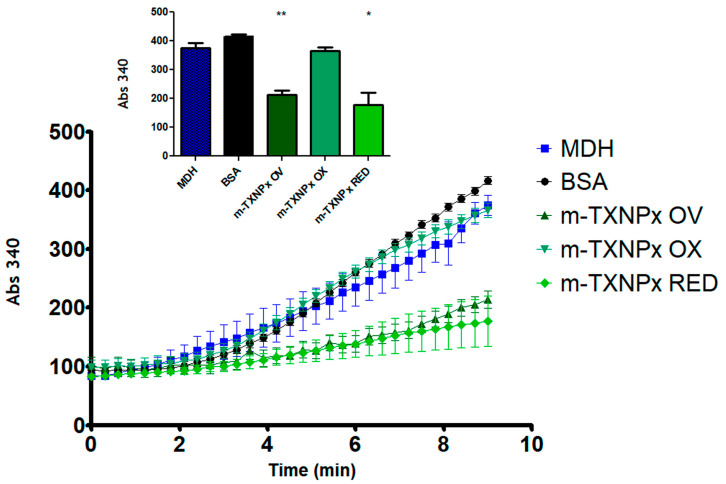
Chaperone activity of m-TXNPx under different redox conditions. The chaperone activity of reduced (RED), oxidized (OX), and overoxidized (OV) m-TXNPx was measured by the temperature-induced aggregation of malate dehydrogenase (MDH). The effect of m-TXNPx (10 µM) on the prevention of thermal aggregation of MDH (1 µM) was monitored at 43 °C for 10 min. The effect of bovine serum albumin (BSA) (10 µM) on the MDH aggregation was used as a negative control. Inset: the significance of the last point at the end of the kinetics was calculated by *t* test, * *p* < 0.05, ** *p* < 0.01. An average of three independent experiments is shown.

We have previously shown that c-TXNPx can prevent the thermal aggregation of MDH in vitro [9]. Here, we wondered whether m-TXNPx has molecular chaperone activity and how this is influenced by the redox state of the protein. We also wanted to compare chaperone activity of m-TXNPx with that of c-TXNPx, previously described in [9], considering that they have different oligomerization profiles, as shown above (Figure 1). Therefore, we analyzed the chaperone capacity of m-TXNPx using recombinant proteins under reduction, oxidation, and overoxidation conditions. As shown in Figure 2, m-TXNPx was able to prevent MDH aggregation, but unlike c-TXNPx, whose activity has been shown to be treatment-independent, in the case of m-TXNPx, the reduced and overoxidized forms showed activity while the oxidized form did not. These results, together with our gel filtration results, demonstrate that m-TXNPx has chaperone activity that is dependent on oxidation status and that the presence of the decameric form of m-TXNPx is not sufficient for this activity.

### 3.3. c-TXNPx and m-TXNPx Show Differences in Susceptibility to Overoxidation during the Catalytic Cycle

**Figure 3:**
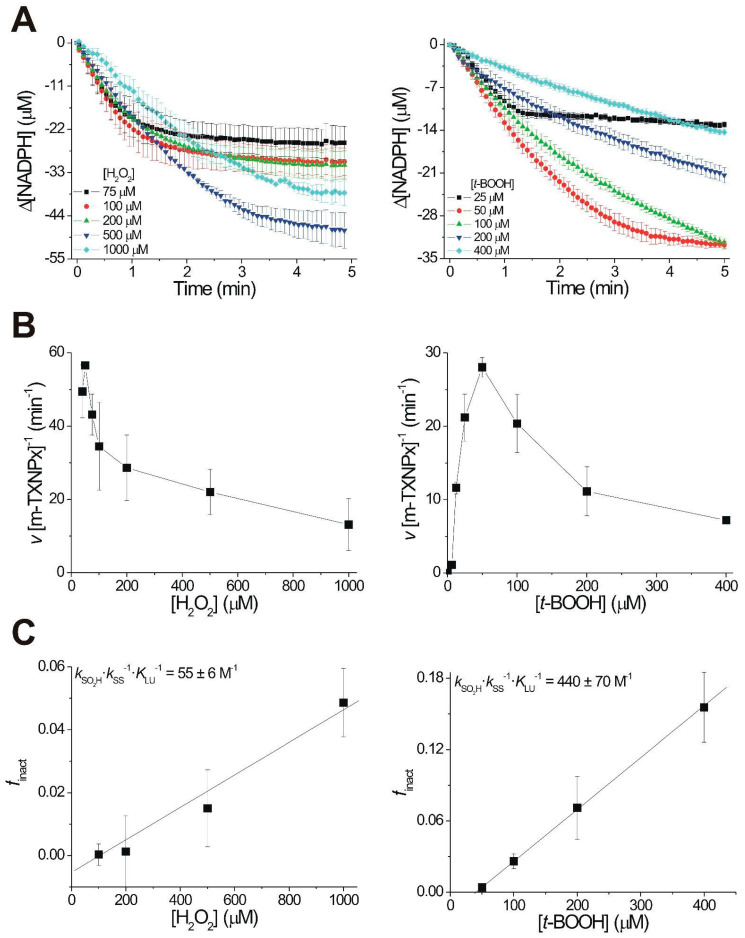
H_2_O_2_-dependent turnover inactivation of m-TXNPx with H_2_O_2_ and *t*-BOOH. Analysis with H_2_O_2,_ and *t*-BOOH. (**A**) Time course of NADPH oxidation at different substrate concentrations. Data averaged from three independent experiments are shown. (**B**) Inactivation kinetics of m-TXNPx. Reactions were performed in the presence of 2 µM *T. cruzi* TR, 5 µM *T. cruzi* TXN 1, 50 µM T(SH)_2_, 0.5 µM m-TXNPx, and 40–1000 µM H_2_O_2_ or 25–400 µM *t*-BOOH. The inactivation kinetic parameters were determined by monitoring NADPH consumption at 340 nm. All reactions were performed at pH 7.4 and 25 °C. The C_hyp1%_ (the peroxide concentration at which 1 out of every 100 Prx molecules is inactivated per turnover) was calculated according to Nelson et al. [14]. (**C**) Replot of *f*_inact_ (inactivated fraction of TXNPx per turnover) as a function of the H_2_O_2_ or *t*-BOOH concentration. The linear fit provides an estimate of the *k*_SO2H_ *k*_SS_^−1^ *K*_LU_^−1^ relation. The details of the analyses are described in [9].

In order to compare the susceptibility to overoxidation between cytosolic and mitochondrial Prxs, we used the coupled peroxidase assay containing NADPH, trypanothione reductase, tryparedoxin, and trypanothione, in the presence of different concentrations of H_2_O_2_ or *t*-BOOH to determine the specific activity, initial rate, and overoxidation index (*f inact*) of TXNPx. In this way, the Prx activity is assayed as it occurs physiologically, as it has all the system components. Compared to the C_hyp1%_ previously estimated for c-TXNPx, m-TXNPx was found to be more sensitive to both peroxides, being approximately 1.2 times more sensitive to H_2_O_2_ and 3 times more sensitive to *t*-BOOH (Figure 3 and Table 1). These results revealed that there are differences in the susceptibility to overoxidation between these two proteins. Both *T. cruzi* Prxs are more robust than human Prx1. *T. cruzi* mitochondrial Prx showed similar overoxidation sensitivity as the human mitochondrial Prx (Prx3).

### 3.4. In Vivo Oligomerization of Prxs in Parasites Treated with Oxidants

**Figure 4:**
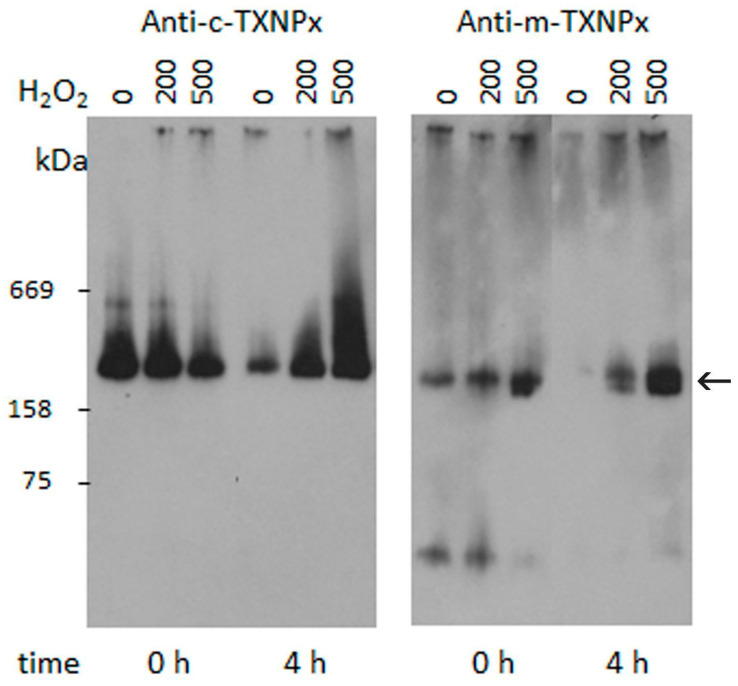
In vivo detection of *T. cruzi* Prxs subjected to oxidative treatment. Exponentially growing epimastigotes were incubated in PBS-1% glucose in the presence of two concentrations of H_2_O_2_ (200 and 500 μM) and then incubated for 0 or 4 h at 28 °C. Parasites were lysed under native conditions and the oligomeric state was analyzed via native PAGE followed by Western blot using anti c-TXNPx and anti-m-TXNPx antibodies. The arrow points to the decamer of both Prx.

We investigated the oligomerization dynamics of c-TXNPx and m-TXNPx in parasites treated with H_2_O_2_ under native conditions. For this purpose, we treated epimastigotes with two different concentrations of H_2_O_2_ (200 and 500 μM) for 30 min, and then suspended the parasites in fresh media to allow recovery. Cell lysates were collected immediately or after 4 h and subjected to native-PAGE and immunoblotting using anti-Prxs antibodies. As shown in Figure 4 and Supplementary Figure S2, at 4 h c-TXNPx in extracts from untreated epimastigotes was mainly present as decameric structures, whereas exposure to H_2_O_2_ converted Prx into HMM complexes in a dose-dependent manner. Interestingly, four hours after the removal of H_2_O_2_ from the medium, the level of c-TXNPx HMM complexes increased with respect to the initial level. Conversely, m-TXNPx was detected as a decamer but also as LMM structures in control and H_2_O_2_ treated-epimastigotes (Figure 4).

### 3.5. Overoxidized Prx Does Not Actively Reverse in Oxidant-Treated Parasites

**Figure 5:**
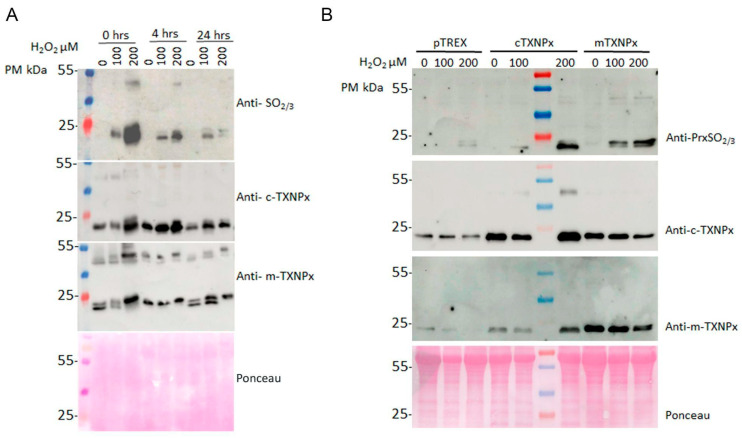

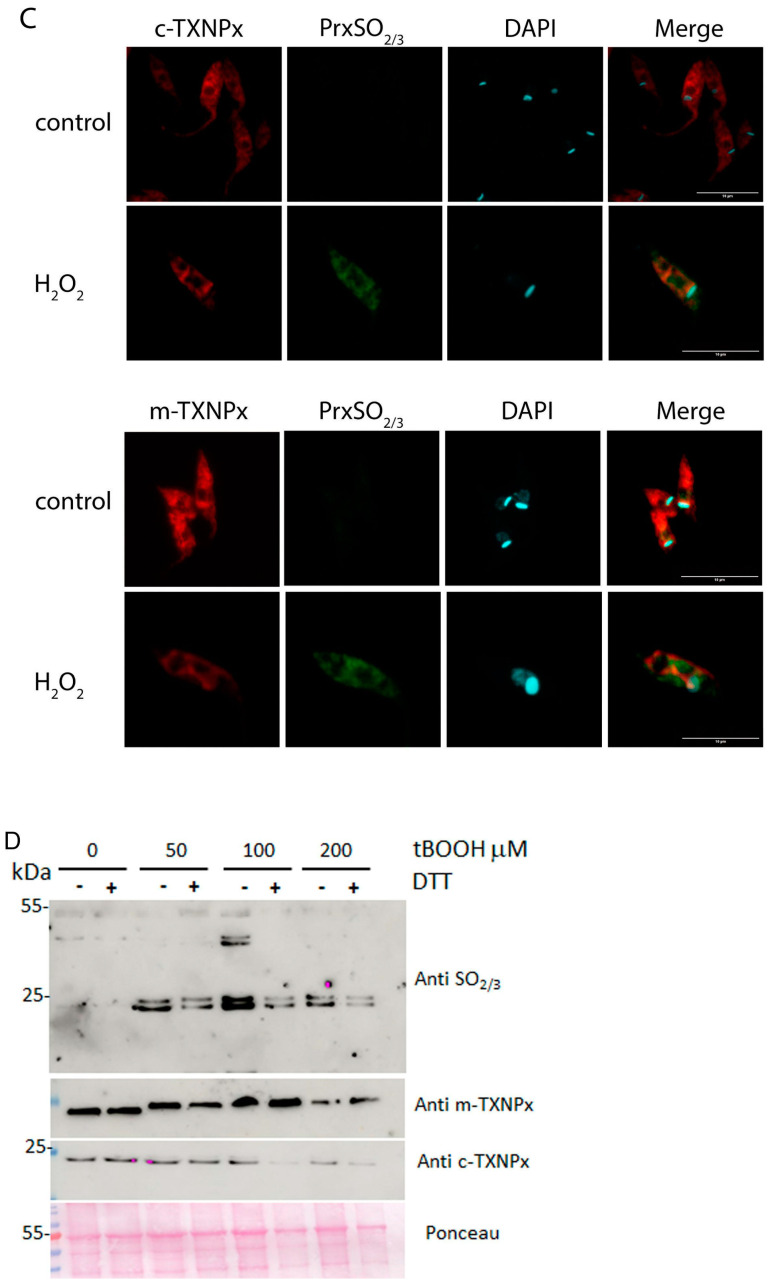
Analysis of the dynamics of overoxidation over time in parasites. (**A**) Wild-type epimastigotes were treated with 100 and 200 μM H_2_O_2_ for 30 min in PBS-1% glucose. Parasites were lysed at different times (0, 4, and 24 h) after growth in LIT medium and analyzed using Western Blot with anti-SO_2/3_ (1/1000) and 15 μg of each extract. The membrane was stripped and analyzed for other antibodies: anti-c-TXNPx (1/5000), and anti-m-TXNPx (1/1000). The loading control was performed via Ponceau-S red staining of the membrane. (**B**) Control line (pTREX) and overexpressing c-TXNPx and m-TXNPx lines were treated with 100 and 200 μM H_2_O_2_ for 30 min in PBS-1% glucose, lysed after growth in LIT medium for 4 h, and analyzed using Western blot. (**C**) Immunofluorescence of oxidant-treated epimastigotes. Epimastigotes (1 × 10^7^) were exposed to PBS 1% glucose (control condition) or 500 µM H_2_O_2_ in PBS 1% glucose for 30 min at 28 °C. After treatment, the parasites were washed and fixed for indirect immunofluorescence assay (IIF). IIF was performed using mouse anti-c-TXNPx (1/200) (**upper left**) or mouse anti-mTXNPx (1/200) (**lower left**) in combination with a rabbit anti-PrxSO_2/3_ (1/1000) antibody (AB Frontiers). The scale bar corresponds to 10 μm. (**D**) Cell-derived trypomastigotes were treated with different concentrations of *t*-BOOH (0, 50, 100, and 200 μM) for 30 min in PBS. The cells were lysed and analyzed as described for the epimastigotes.

Next, we investigated whether c-TXNPx and m-TXNPx are overoxidized in parasites treated with H_2_O_2_ and wondered how long the overoxidized forms of these proteins persist after oxidative stress. To address this question, we used oxidant-treated epimastigotes to evaluate the overoxidation of Prx and its recovery after 4 and 24 h. After treatment with 100 or 200 μM H_2_O_2_ for 30 min, the epimastigotes were washed and incubated in LIT medium for 4 and 24 h. At each time point, protein extracts were obtained and resolved by Western blotting using SDS-PAGE. As shown in Figure 5A, there is overoxidation in the epimastigotes treated with H_2_O_2_, and the signal is bigger at the higher concentration of the oxidant. The overoxidation signal was still detectable 24 h after treatment. The signal seems to correspond to both cytosolic and mitochondrial Prxs, evidenced by the difference in size of both proteins. Next, we wondered whether higher Prx levels in parasites could protect against overoxidation, so we used Prx-overexpressing lines. Overoxidation at both concentrations of H_2_O_2_ at all times studied was observed, with a greater signal in the Prx-overexpressing lines compared to the control line, in agreement with the increased level of Prx expression in these lines (Figure 5B and Supplementary Figure S3). To study the subcellular distribution of the oxidized forms of Prxs, the epimastigotes were treated with 500 μM H_2_O_2_ for 30 min and visualized via indirect immunofluorescence using anti-c-TXNPx or anti-m-TXNPx and anti-PrxSO_2/3_ antibodies. c-TXNPx is located throughout the cytoplasm and most cytosolic proteins are overoxidized after oxidative treatment (Figure 5C and Supplementary Figure S4). However, only a fraction of the label corresponding to m-TXNPx overlapped with the label revealed by the anti- Prx-SO_2/3_ antibody. Since it is the trypomastigote form that faces oxidative stress in the infective process of mammalian cells, we also analyzed whether Prxs were overoxidized in cell-derived trypomastigotes incubated with 50, 100, and 200 μM *t*-BOOH. We detected, using Western blot, overoxidation at all concentrations studied (Figure 5D).

### 3.6. c-TXNPx and m-TXNPx Are Physiologically Overoxidized in Infection

**Figure 6:**
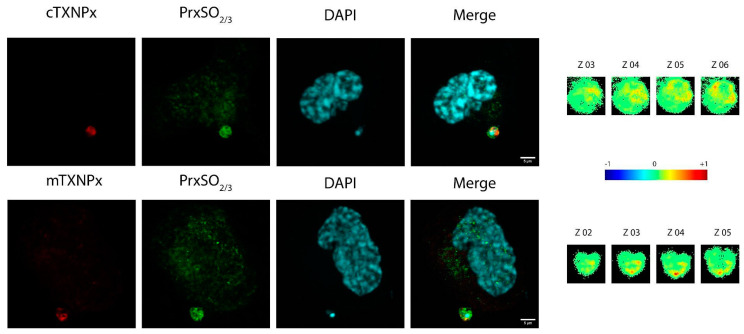
Immunofluorescence of amastigotes after infection of THP1 cells. Differentiated THP1 were seeded in 12-well plates (1 × 10^5^) and infected with trypomastigotes (MOI 5:1) for 2 h. After the interaction, cells were fixed, and immunofluorescence was performed using a mouse anti-c-TXNPx (1/200) (**upper right**) or mouse anti-mTXNPx (1/200) (**lower right**) in combination with a rabbit anti-PrxSO_2/3_ antibody (AB Frontiers). Nuclear and mitochondrial DNA was stained with DAPI. Confocal images were analyzed using Fiji (https://imagej.net/Fiji, accessed on 22 August 2023). Colocalization colormaps and coloc2 plugin were used for colocalization analysis. M1 and M2 (Manders’ coefficients) indicate the proportion of overlap between the two channels. Pearson’s coefficient indicates the correlation between the two channels. The scale bar corresponds to 5 μm.

Next, we aimed to analyze the overoxidation of c-TXNPx and m-TXNPx in the context of infection, as a consequence of the physiological generation of cellular oxidants during infection. Immunofluorescence analysis was performed in the absence of exogenously added oxidants on in vitro *T. cruzi* infected macrophage cultures labeled with specific anti-Prx and anti-Prx-SO_2/3_ antibodies. At 2 h post infection, we found labeling of intracellular amastigotes with anti-Prx-SO_2/3_ antibody as well as partial colocalization with c-TXNPx and m-TXNPx antibodies (Figure 6 and Supplementary Figure S5). The Prx-SO_2/3_ labeling was no longer visible after 48 h of infection. A greater correlation between the labels was found for c-TXNPx than for m-TXNPx. Pearson’s R for c-TXNPx and PrxSO_2/3_ was 0.59 ± 0.097, and the percentage of c-TXNPx colocalized with sulfinic acid (tM1) was 95.7% ± 3%. On the other hand, Pearson’s R for m-TXNPx and PrxSO_2/3_ was lower and more variable (0.29 ± 0.25), while 56.2% ± 18.6% of m-TXNPx was colocalized with PrxSO_2/3_ label (tM1). The percentage of PrxSO_2/3_ that colocalized with c-TXNPx (tM2) was 75% ± 11%, and with m-TXNPx (tM2) it was 66.4% ± 0.18%. These results demonstrate that Prx overoxidation occurs in a physiological context during the infection process. Although the endogenous source of ROS in the host cells remains to be determined, we can speculate that it is probably due to the assembly of the Nox2 enzyme and its activation inside the phagosome, which has been described as occurring in activated macrophages.

### 3.7. Oxidant Treatment Alters Parasite Infectivity

**Figure 7:**
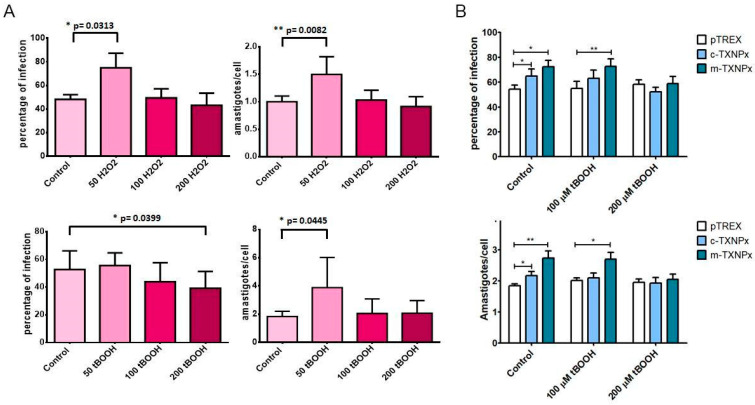
Infectivity of oxidant-treated trypomastigotes. Wild-type cell-derived trypomastigotes (**A**) or trypomastigotes from control pTREX, c-TXNPx-, and m-TXNPx-overexpressing lines (**B**) were treated with different concentrations of H_2_O_2_ or *t*-BOOH in PBS for 30 min and used in in vitro cell infection experiments. Vero cells were seeded in 12-well plates (1 × 10^5^) and infected with trypomastigotes treated with oxidants (MOI 5:1) for 48 h. After interaction, cells were fixed and stained with DAPI. The number of infected cells and amastigotes per infected cell was determined by counting the nuclei of Vero cells and the kinetoplast of amastigotes using epifluorescence microscopy. Analysis was performed using the Icy software. Statistical analysis of three independent experiments was performed using an unpaired *t*-test with *p* < 0.05 considered significant (* *p* < 0.05; ** *p* < 0.01).

Since we observed that Prxs in trypomastigotes become overoxidized after oxidant treatment and during the infection of phagocytic cells, we investigated how the redox state affects the infectivity capacity of trypomastigotes. Therefore, we performed in vitro infection assays using trypomastigotes previously treated with 0, 50, 100, or 200 μM H_2_O_2_ or *t*-BOOH. Two parameters were evaluated at 48 h after infection: the percentage of infection and the average number of amastigotes per cell. Parasites were affected differently by both oxidants. Parasites treated with *t*-BOOH (at 100 or 200 μM) showed a decrease in their capacity for infection (Figure 7A) but not in their proliferation capacity. In contrast, H_2_O_2_ treatment at the same concentrations did not affect trypomastigote infectivity. Rather, 50 μM H_2_O_2_ treatment promoted infectivity (Figure 7A). Interestingly, both oxidants at lower concentrations (50 μM) promoted amastigote proliferation. These results showed that low concentrations of H_2_O_2_ generate a higher infection capacity and amastigote proliferation, probably related to a possible role of H_2_O_2_ as a redox signaling molecule in physiological processes. As these results did not allow us to assess the possible role of Prx in this oxidative context, we performed infection experiments as described above using c-TXNPx- and m-TXNPx-overexpressing lines and compared them with their respective controls. As previously described in [5], we observed that in the absence of oxidant treatment, overexpressing lines for both Prxs were more infective than the control (Figure 7B). However, there were no significant differences in infectivity when trypomastigotes from these overexpressing lines were treated with 200 μM *t*-BOOH compared to the control line. Interestingly, only at 100 μM *t*-BOOH the overexpressing lines tended to be more infective than the control, and only the m-TXNPx-overexpressing line showed significant differences compared to the control (Figure 7B). All these results indicate that the oxidative status of Prxs can alter their function and that this affects the ability to promote infectivity.

### 3.8. T. cruzi Prxs Contribute to the Acquisition of Cellular Thermotolerance

**Figure 8:**
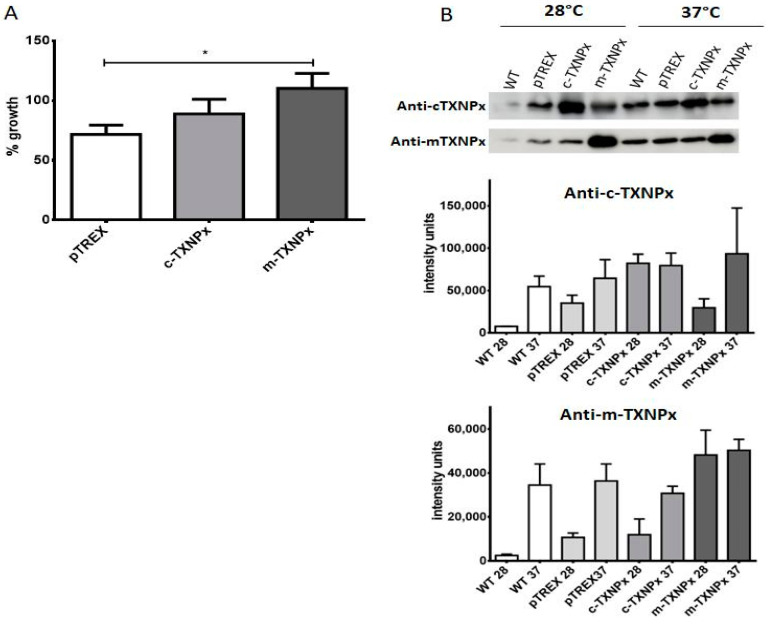
Thermotolerance of Prx-overexpressing lines. (**A**) Parasites were grown at 28 °C or 37 °C for 72 h. The percentage of growth at 37 °C with respect to 28 °C was estimated for each parasite line. (**B**) Proteins were extracted and analyzed via Western blot using the specific Prx antibodies. Above, a representative Western blot experiment. Below, gels from two independent experiments were quantified using Image J software (version 1.52p). The intensity units of each condition were plotted after normalization to the intensity values of tubulin used as a loading control. * *p* < 0.05.

Since *T. cruzi* parasites are not only exposed to oxidative stress but also to changing temperature conditions during their life cycle, and since we have observed that c-TXNPx and m-TXNPx can prevent the thermal aggregation of MDH in vitro, we investigated whether *T. cruzi* Prxs play a role in the adaptation and survival of this parasite against temperature changes. To this end, we tested whether c-TXNPx and m-TXNPx could confer thermotolerance by incubating epimastigotes overexpressing both Prxs at 37 °C for 72 h and comparing growth with that of parasites incubated at 28 °C. As shown in Figure 8A, overexpression of m-TXNPx conferred thermotolerance (approximately 50% compared to the pTREX control line). A Western blot analysis of extracts from the thermotolerant parasites showed that under these conditions, both c-TXNPx and m-TXNPx proteins were strongly induced at 37 °C compared to growth at 28 °C. Remarkably, induction was better observed in extracts from non-overexpressing lines such as wild-type, vector pTREX control parasites, and in the case of overexpressing transfectant lines, this induction is seen for the other Prx that is not overexpressed (Figure 8B and Supplementary Figure S6).

## 4. Discussion

In various organisms, the functions of Prxs have been shown to depend on their oligomerization and oxidation states. In some cases, overoxidation favors the formation of HMM complexes, leading to a functional change in these enzymes from peroxidase to chaperone activity [20]. In this study, we aimed to investigate the oxidation and oligomerization dynamics of *T. cruzi* cytosolic and mitochondrial Prxs, to better understand their role in the parasite life cycle and infection process. Our findings reveal differences between *T. cruzi* cytosolic and mitochondrial Prxs in terms of redox-dependent oligomerization, chaperone activity, and susceptibility to overoxidation, potentially contributing to functional distinctions between these two proteins.

In terms of oligomerization, H_2_O_2_ treatment of recombinant proteins under overoxidation conditions produced both decameric and HMM forms of c-TXNPx, whereas in the case of m-TXNPx, a similar treatment produced mainly decamers and oligomers of LMM, compatible with a tetramer or dimer. This difference was also seen in cultured epimastigotes treated with H_2_O_2_. This behavior is shared with mitochondrial Prx from other parasites like *L. braziliensis* and *L. infantum*, which suffer from decamer to dimer transition under different conditions, such as changes in pH or oxidized conditions [21,22,23].

Regarding overoxidation, m-TXNPx is approximately 1.2 times more sensitive to overoxidation than c-TXNPx, but twice as robust as human Prx1, in the same order as human mitochondrial Prx3, and almost five times more robust than *Leptospira interrogans* alkyl hydroperoxide reductase C (*Li*AhpC). Both *T. cruzi* cytosolic and mitochondrial Prxs contain the structural elements present in most sensitive Prxs, the “GGLG” and “YF’’ motifs, making them susceptible to overoxidation [24,25,26,27]. Nevertheless, susceptibility varies in eukaryotic Prxs. For example, although both human Prx2 and Prx3 have the GGLG and YF motifs, mitochondrial Prx3 is 10-fold more robust than Prx2 [19,28,29]. Two other sequence motifs, motif A, i.e., D*X*_8_(N/G)*X*_10_H*X*_27_(S/G), and motif B, i.e., T*X*_3_(S/T) [19], may influence susceptibility to overoxidation. These motifs are absent in *T. cruzi* c-TXNPx, whereas m-TXNPx has motif A with a Gln instead of a His and lacks motif B. The presence of Gln in motif A, as seen in sensitive Prxs like human Prx2, may explain the differences in susceptibility to overoxidation between the two *T. cruzi* enzymes. Further mutant studies are needed to confirm these structural elements’ relevance in *T. cruzi* Prx.

Here, we have shown that both *T. cruzi* proteins can undergo overoxidation when exposed to exogenous oxidants in cultured parasites, including both epimastigotes and cell-derived trypomastigotes. Treatment with H_2_O_2_ in epimastigotes leads to overoxidation, resulting in monomeric and dimeric overoxidized species in SDS-PAGE under reducing conditions. This differs from *T. brucei* Prxs, where H_2_O_2_-induced overoxidation in the procyclic form produces only overoxidized monomeric species [30]. Considering that the Prxs from *T. cruzi* and *T. brucei* share a high degree of sequence similarity (ranging from 76% to 84% sequence identity), the differences observed could be attributed to structural variations in the proteins and distinct dynamics in the overoxidation process.

For some Prxs, the overoxidation can be reversed through an ATP-dependent process catalyzed by an enzyme called sulfiredoxin (Srx) [31,32]. In many eukaryotic Prxs, reversion of the overoxidized signal has been observed at 3 h after treatment due to the activity of Srx [32,33]. Here, we showed that overoxidation in epimastigotes increases with time, does not reverse in the short term, and persists for at least 24 h after treatment, indicating that these forms are not actively reversible and are likely to slowly disappear as the proteins are degraded. This finding aligns with the absence of Srx activity and an active reversion process in *T. cruzi*.

Here, we showed that c-TXNPx and m-TXNPx are physiologically overoxidized during infection, as evidenced by the presence of the signal in intracellular amastigotes within infected cells. It should be noted that the concentration of H_2_O_2_ in phagolysosomes can reach high values of 100 μM [34]. Therefore, we reasoned that at least part of the protein should become overoxidized in this scenario. Our results show that the generation of reactive oxygen species within the phagosome, where the parasite initially resides after internalization, can lead to the overoxidation of *T. cruzi* Prxs. These results have led us to speculate that Prxs, even when overoxidized, might have an important role in the infection process by acting as molecular chaperones and protecting parasites from heat stress.

To further investigate the role of Prxs during infection, we used mitochondrial and cytosolic Prxs-overexpressing lines to test their protective effect against H_2_O_2_-induced overoxidation. We found that an increase in protein levels did not protect against overoxidation. In fact, in both overexpressing lines, we observed a greater signal of overoxidation at higher doses of the oxidant. This signal persisted for 24 h after treatment, similar to the wild-type line. This differs from previous findings in *T. cruzi* epimastigotes overexpressing c-TXNPx and treated with peroxynitrite, where a much weaker overoxidation signal was reported [7]. The differences observed may be attributed to variations in the level of protein overexpression in the transfectant lines or differences in the oxidants used in the experiments.

Previously, we demonstrated that the overexpression of *T. cruzi* TXNPx is associated with an increase in parasite infectivity [5]. In our in vitro infection experiments using oxidant-treated trypomastigotes, we found that treatment with the highest doses of oxidant affected infection capacity. Furthermore, trypomastigotes from overexpressing lines, when treated with oxidants, did not display higher infectivity than controls. These results collectively suggest that the oxidative status of Prxs plays an important role in the infection process. During the infection process, parasites encounter oxidative species that lead to some degree of Prx overoxidation. However, the high expression of Prx likely compensates for the portion that becomes inactivated, thus ensuring successful infection. However, in cases where a larger proportion of Prx becomes overoxidized, as seen in our experiments with treated trypomastigotes, it compromises infection capacity. This suggests that the reduced form of Prxs with peroxidase activity is crucial for the infection process. This is consistent with previous results where the inhibition of the peroxidase activity of *T. cruzi* m-TXNPx, but not its chaperone activity, reduced infection in macrophages [10].

Considering that parasites also encounter elevated temperatures during the early stages of infection, we examined the expression of Prxs in parasites grown at 37 °C. Our Western blot analysis showed higher levels of Prx expression at 37 °C compared to 28 °C. Presumably, the heat-induced increase in these proteins leads to greater temperature resistance in control lines compared to overexpressing lines that already have elevated Prx levels. Our in vivo thermotolerance assay results suggest that parasite Prxs play a critical role not only in promoting cell survival under oxidative stress conditions but also during temperature fluctuations encountered by parasites as they invade mammalian hosts during the infection cycle. In other parasites, Prxs are involved in temperature adaptation and are essential for thermotolerance, as observed in *L. infantum* and *L. braziliensis* m-TXNPx [22,35].

We and others have previously shown that cytosolic and mitochondrial *T. cruzi* Prxs have chaperone activities [9,10]. In this study, we show that *T. cruzi* m-TXNPx has chaperone holdase activity, preventing protein aggregation. Additionally, Specker et al. (2022) showed that *T. cruzi* m-TXNPx can enhance the refolding of green fluorescent protein (GFP) in both the reduced and oxidized states. In this assay, GFP is denatured with HCl and refolding in an ATP-free buffer is monitored by fluorescence, which is performed at a 1:100 molar ratio of GFP to m-TXNPx. This suggests a potential foldase activity of m-TXNPx independent of ATP hydrolysis. While holdases prevent protein aggregation under stress conditions by binding tightly to proteins, ATP-dependent chaperone foldases require ATP binding and hydrolysis to assist in protein folding. There are many reports of 2-Cys Prxs from different organisms becoming inactive peroxidases and active ATP-independent chaperones that prevent protein aggregation during oxidative, acidic, and temperature stress [36]. Our results, coupled with those from Specker et al., suggest that m-TXNPx may possess both holdase and ATP-independent foldase activities. There is only one report of a 2-Cys Prx with both chaperone holdase and ATP-dependent foldase activity, the 2-Cys Prx from the plant *Arabidopsis thaliana* [37]. Additionally, there are two ATP-independent foldases known to guide substrate proteins in correct folding via an ATP-free mechanism, although they differ structurally from m-TXNP [38,39]. Further studies are needed to characterize the chaperone activity of m-TXNPx with other substrates, with or without ATP, and with different chaperone-to-target protein ratios, as a molar ratio of 1 to 100 raises doubts as to whether this effect is physiologically relevant.

*T. cruzi* m-TXNPx exhibits chaperone activity in its reduced and overoxidized states but has no activity when oxidized, indicating that the reduced decameric form alone is sufficient for chaperone function. This differs from mammalian Prxs, which require HMM oligomers for this activity. Other mitochondrial parasitic Prxs also display chaperone activities. Reduced but not oxidized m-TXNPx from *L. infantum* exhibits chaperone activity induced by temperature, but does not have foldase activity; this activity is crucial for host virulence and thermotolerance in the insect form of the parasite [22]. *L. braziliensis* m-TXNPx displays chaperone activity in both its oxidized and reduced decameric states, contributing to thermotolerance in parasites [35]. These results illustrate not only the distinct behavior of parasite Prxs compared to thei mammalian counterparts, but also the structural and functional differences among different trypanosomatid species.

Several roles have been proposed for Prx overoxidation in cells. It has been described that Prx overoxidation allows local increases in hydrogen peroxide, leading to cellular signaling processes [40,41]. In addition, Prxs have been described to interact with other proteins, modifying their redox status and regulating their function [42,43]. The involvement of parasite Prxs overoxidation in H_2_O_2_ signaling remains to be established. Alternatively, overoxidation may enhance the chaperone function of these proteins, aiding in combating protein-unfolding conditions. In yeast, overoxidation of Prx has been proposed to play a role in maintaining a reduced pool of thioredoxin available to reduce other oxidized proteins under stress conditions [44]. In *T. cruzi*, thioredoxin activity is carried out by tryparedoxin, which reduces Prx disulfides. Interestingly, the interactomes of *T. cruzi* tryparedoxin 1 and 2 have been described, revealing interactions with numerous proteins involved in vital cellular activities such as oxidative, protein, and energy metabolism [45,46,47]. In this context, it is tempting to speculate that the sensitivity of *T. cruzi* Prxs to overoxidation has provided an adaptive advantage by preserving the reducing capacity of tryparedoxins, as has been proposed for *Saccharomyces cerevisiae* [44]. Importantly, tryparedoxin has been shown to exert significant control over peroxide reduction flux, underscoring its crucial role in ensuring parasite viability [48].

## 5. Conclusions

In summary, we investigated the oxidation and oligomerization dynamics of *T. cruzi* cytosolic and mitochondrial Prxs. This work shows that *T. cruzi* cytosolic and mitochondrial Prxs differ in their redox-dependent oligomerization, chaperone activity, and susceptibility to overoxidation. We observed that *T. cruzi* Prxs were overoxidized in vivo and in vitro, with m-TXNPx being a little more sensitive to overoxidation than c-TXNPx. We also demonstrated that *T. cruzi* m-TXNPx has chaperone holdase activity that is dependent on the redox state. We observed that both epimastigote and trypomastigote parasites exhibit Prx overoxidation when treated with H_2_O_2_. We also showed that overoxidation of Prxs occurs in the context of the infection process. Overexpression of Prxs does not protect against overoxidation. Our results suggest that parasitic Prxs are induced under temperature changes, providing some thermotolerance for cell survival. Structural and functional changes in *T. cruzi* Prx may be relevant during the infection process, aiding in the parasite’s survival under varying conditions. These studies enhance our understanding of these proteins as potential virulence factors.

## Figures and Tables

**Table 1:** Determination of the C_Hyp1%_ (µM) value of m-TXNPx with *t*-BOOH and H_2_O_2_. Comparison with the values of other Prxs. ^¤^ [9]; * [14]; ^#^ [18]; ^&^ [19].

Peroxide	Enzyme	C_Hyp1%_ (mM)
H_2_O_2_	m-TXNPx	180 ± 20
	c-TXNPx	210 ± 20 ^¤^
	Prx1	62 *
	Prx3	127 ^&^
	*Lin*AhpC	30 ± 2 ^#^
*t*-BOOH	m-TXNPx	23 ± 4
	c-TXNPx	35 ± 4
	*Lin*AhpC	22 ± 1 ^#^

## Data Availability

Data sharing not applicable.

## Supplementary Materials

The following supporting information can be downloaded at: https://www.mdpi.com/article/10.3390/pathogens12101273/s1, Figure S1: Loading control of Western blots shown in Figure 1A; Figure S2: Loading control of Western blots shown in Figure 4; Figure S3: Analysis of the dynamics of overoxidation over time in epimastigotes; Figure S4: Immunofluorescence of epimastigotes; Figure S5: Immunofluorescence of THP1 macrophages infected with trypomastigotes and fixed after internalization; Figure S6: Loading control of Western blot shown in Figure 8B.

## References

[1] Pérez-Molina J.A., Molina I. (2018). Chagas Disease. Lancet Lond. Engl..

[2] Palmisano G., Yoshida N. (2023). Editorial: World Chagas Disease Day 2022. Front. Cell. Infect. Microbiol..

[3] Piñeyro M.D., Arcari T., Robello C., Radi R., Trujillo M. (2011). Tryparedoxin Peroxidases from Trypanosoma Cruzi: High Efficiency in the Catalytic Elimination of Hydrogen Peroxide and Peroxynitrite. Arch. Biochem. Biophys..

[4] Wilkinson S.R., Horn D., Prathalingam S.R., Kelly J.M. (2003). RNA Interference Identifies Two Hydroperoxide Metabolizing Enzymes That Are Essential to the Bloodstream Form of the African Trypanosome. J. Biol. Chem..

[5] Piñeyro M.D., Parodi-Talice A., Arcari T., Robello C. (2008). Peroxiredoxins from Trypanosoma Cruzi: Virulence Factors and Drug Targets for Treatment of Chagas Disease?. Gene.

[6] Iyer J.P., Kaprakkaden A., Choudhary M.L., Shaha C. (2008). Crucial Role of Cytosolic Tryparedoxin Peroxidase in Leishmania Donovani Survival, Drug Response and Virulence. Mol. Microbiol..

[7] Piacenza L., Peluffo G., Alvarez M.N., Kelly J.M., Wilkinson S.R., Radi R. (2008). Peroxiredoxins Play a Major Role in Protecting Trypanosoma Cruzi against Macrophage- and Endogenously-Derived Peroxynitrite. Biochem. J..

[8] Vallières C., Golinelli-Cohen M.-P., Guittet O., Lepoivre M., Huang M.-E., Vernis L. (2023). Redox-Based Strategies against Infections by Eukaryotic Pathogens. Genes.

[9] Piñeyro M.D., Arias D., Ricciardi A., Robello C., Parodi-Talice A. (2019). Oligomerization Dynamics and Functionality of Trypanosoma Cruzi Cytosolic Tryparedoxin Peroxidase as Peroxidase and Molecular Chaperone. Biochim. Biophys. Acta Gen. Subj..

[10] Specker G., Estrada D., Radi R., Piacenza L. (2022). Trypanosoma Cruzi Mitochondrial Peroxiredoxin Promotes Infectivity in Macrophages and Attenuates Nifurtimox Toxicity. Front. Cell. Infect. Microbiol..

[11] Castro H., Teixeira F., Romao S., Santos M., Cruz T., Flórido M., Appelberg R., Oliveira P., Ferreira-da-Silva F., Tomás A.M. (2011). Leishmania Mitochondrial Peroxiredoxin Plays a Crucial Peroxidase-Unrelated Role during Infection: Insight into Its Novel Chaperone Activity. PLoS Pathog..

[12] Yang K.-S., Kang S.W., Woo H.A., Hwang S.C., Chae H.Z., Kim K., Rhee S.G. (2002). Inactivation of Human Peroxiredoxin I during Catalysis as the Result of the Oxidation of the Catalytic Site Cysteine to Cysteine-Sulfinic Acid. J. Biol. Chem..

[13] Lorenzi H.A., Vazquez M.P., Levin M.J. (2003). Integration of Expression Vectors into the Ribosomal Locus of Trypanosoma Cruzi. Gene.

[14] Nelson K.J., Parsonage D., Karplus P.A., Poole L.B. (2013). Evaluating Peroxiredoxin Sensitivity Toward Inactivation by Peroxide Substrates. Methods in Enzymology.

[15] Bolte S., Cordelières F.P. (2006). A Guided Tour into Subcellular Colocalization Analysis in Light Microscopy. J. Microsc..

[16] Rolón M., Vega C., Escario J.A., Gómez-Barrio A. (2006). Development of Resazurin Microtiter Assay for Drug Sensibility Testing of Trypanosoma Cruzi Epimastigotes. Parasitol. Res..

[17] de Chaumont F., Dallongeville S., Chenouard N., Hervé N., Pop S., Provoost T., Meas-Yedid V., Pankajakshan P., Lecomte T., Le Montagner Y. (2012). Icy: An Open Bioimage Informatics Platform for Extended Reproducible Research. Nat. Methods.

[18] Arias D.G., Reinoso A., Sasoni N., Hartman M.D., Iglesias A.A., Guerrero S.A. (2014). Kinetic and Structural Characterization of a Typical 2-Cysteine Peroxiredoxin from Leptospira Interrogans Exhibiting Redox Sensitivity. Free. Radic. Biol. Med..

[19] Bolduc J.A., Nelson K.J., Haynes A.C., Lee J., Reisz J.A., Graff A.H., Clodfelter J.E., Parsonage D., Poole L.B., Furdui C.M. (2018). Novel Hyperoxidation Resistance Motifs in 2-Cys Peroxiredoxins. J. Biol. Chem..

[20] Jang H.H., Lee K.O., Chi Y.H., Jung B.G., Park S.K., Park J.H., Lee J.R., Lee S.S., Moon J.C., Yun J.W. (2004). Two Enzymes in One; Two Yeast Peroxiredoxins Display Oxidative Stress-Dependent Switching from a Peroxidase to a Molecular Chaperone Function. Cell.

[21] Morais M.A.B., Giuseppe P.O., Souza T.A.C.B., Alegria T.G.P., Oliveira M.A., Netto L.E.S., Murakami M.T. (2015). How pH Modulates the Dimer-Decamer Interconversion of 2-Cys Peroxiredoxins from the Prx1 Subfamily. J. Biol. Chem..

[22] Teixeira F., Castro H., Cruz T., Tse E., Koldewey P., Southworth D.R., Tomás A.M., Jakob U. (2015). Mitochondrial Peroxiredoxin Functions as Crucial Chaperone Reservoir in Leishmania Infantum. Proc. Natl. Acad. Sci. USA..

[23] Cao Z., Bhella D., Lindsay J.G. (2007). Reconstitution of the Mitochondrial PrxIII Antioxidant Defence Pathway: General Properties and Factors Affecting PrxIII Activity and Oligomeric State. J. Mol. Biol..

[24] Poynton R.A., Peskin A.V., Haynes A.C., Lowther W.T., Hampton M.B., Winterbourn C.C. (2016). Kinetic Analysis of Structural Influences on the Susceptibility of Peroxiredoxins 2 and 3 to Hyperoxidation. Biochem. J..

[25] Hall A., Nelson K., Poole L.B., Karplus P.A. (2011). Structure-Based Insights into the Catalytic Power and Conformational Dexterity of Peroxiredoxins. Antioxid. Redox Signal..

[26] Koo K.H., Lee S., Jeong S.Y., Kim E.T., Kim H.J., Kim K., Song K., Chae H.Z. (2002). Regulation of Thioredoxin Peroxidase Activity by C-Terminal Truncation. Arch. Biochem. Biophys..

[27] Sayed A.A., Williams D.L. (2004). Biochemical Characterization of 2-Cys Peroxiredoxins from Schistosoma Mansoni. J. Biol. Chem..

[28] Cox A.G., Peskin A.V., Paton L.N., Winterbourn C.C., Hampton M.B. (2009). Redox Potential and Peroxide Reactivity of Human Peroxiredoxin 3. Biochemistry.

[29] Peskin A.V., Dickerhof N., Poynton R.A., Paton L.N., Pace P.E., Hampton M.B., Winterbourn C.C. (2013). Hyperoxidation of Peroxiredoxins 2 and 3: Rate Constants for the Reactions of the Sulfenic Acid of the Peroxidatic Cysteine. J. Biol. Chem..

[30] Bogacz M., Dirdjaja N., Wimmer B., Habich C., Krauth-Siegel R.L. (2020). The Mitochondrial Peroxiredoxin Displays Distinct Roles in Different Developmental Stages of African Trypanosomes. Redox Biol..

[31] Biteau B., Labarre J., Toledano M.B. (2003). ATP-Dependent Reduction of Cysteine-Sulphinic Acid by S. Cerevisiae Sulphiredoxin. Nature.

[32] Woo H.A., Kang S.W., Kim H.K., Yang K.-S., Chae H.Z., Rhee S.G. (2003). Reversible Oxidation of the Active Site Cysteine of Peroxiredoxins to Cysteine Sulfinic Acid. Immunoblot Detection with Antibodies Specific for the Hyperoxidized Cysteine-Containing Sequence. J. Biol. Chem..

[33] Jeong W., Bae S.H., Toledano M.B., Rhee S.G. (2012). Role of Sulfiredoxin as a Regulator of Peroxiredoxin Function and Regulation of Its Expression. Free Radic. Biol. Med..

[34] Melo R.C.N., Fabrino D.L., D’Ávila H., Teixeira H.C., Ferreira A.P. (2003). Production of Hydrogen Peroxide by Peripheral Blood Monocytes and Specific Macrophages during Experimental Infection with Trypanosoma Cruzi in Vivo. Cell Biol. Int..

[35] Morais M.A.B., Giuseppe P.O., Souza T.A.C.B., Castro H., Honorato R.V., Oliveira P.S.L., Netto L.E.S., Tomas A.M., Murakami M.T. (2017). Calcium and Magnesium Ions Modulate the Oligomeric State and Function of Mitochondrial 2-Cys Peroxiredoxins in Leishmania Parasites. J. Biol. Chem..

[36] Ulrich K., Schwappach B., Jakob U. (2021). Thiol-Based Switching Mechanisms of Stress-Sensing Chaperones. Biol. Chem..

[37] Lee E.M., Lee S.S., Tripathi B.N., Jung H.S., Cao G.P., Lee Y., Singh S., Hong S.H., Lee K.W., Lee S.Y. (2015). Site-Directed Mutagenesis Substituting Cysteine for Serine in 2-Cys Peroxiredoxin (2-Cys Prx A) of *Arabidopsis Thaliana* Effectively Improves Its Peroxidase and Chaperone Functions. Ann. Bot..

[38] Mitra R., Gadkari V.V., Meinen B.A., van Mierlo C.P.M., Ruotolo B.T., Bardwell J.C.A. (2021). Mechanism of the Small ATP-Independent Chaperone Spy Is Substrate Specific. Nat. Commun..

[39] He W., Li X., Xue H., Yang Y., Mencius J., Bai L., Zhang J., Xu J., Wu B., Xue Y. (2022). Insights into the Client Protein Release Mechanism of the ATP-Independent Chaperone Spy. Nat. Commun..

[40] Rhee S.G., Woo H.A., Kil I.S., Bae S.H. (2012). Peroxiredoxin Functions as a Peroxidase and a Regulator and Sensor of Local Peroxides. J. Biol. Chem..

[41] Wood Z.A., Poole L.B., Karplus P.A. (2003). Peroxiredoxin Evolution and the Regulation of Hydrogen Peroxide Signaling. Science.

[42] Tachibana T., Okazaki S., Murayama A., Naganuma A., Nomoto A., Kuge S. (2009). A Major Peroxiredoxin-Induced Activation of Yap1 Transcription Factor Is Mediated by Reduction-Sensitive Disulfide Bonds and Reveals a Low Level of Transcriptional Activation. J. Biol. Chem..

[43] Netto L.E.S., Antunes F. (2016). The Roles of Peroxiredoxin and Thioredoxin in Hydrogen Peroxide Sensing and in Signal Transduction. Mol. Cells.

[44] Day A.M., Brown J.D., Taylor S.R., Rand J.D., Morgan B.A., Veal E.A. (2012). Inactivation of a Peroxiredoxin by Hydrogen Peroxide Is Critical for Thioredoxin-Mediated Repair of Oxidized Proteins and Cell Survival. Mol. Cell.

[45] Piñeyro M.D., Parodi-Talice A., Portela M., Arias D.G., Guerrero S.A., Robello C. (2011). Molecular Characterization and Interactome Analysis of Trypanosoma Cruzi Tryparedoxin 1. J. Proteom..

[46] Arias D.G., Piñeyro M.D., Iglesias A.A., Guerrero S.A., Robello C. (2015). Molecular Characterization and Interactome Analysis of Trypanosoma Cruzi Tryparedoxin II. J. Proteom..

[47] Dias L., Peloso E.F., Leme A.F.P., Carnielli C.M., Pereira C.N., Werneck C.C., Guerrero S., Gadelha F.R. (2018). Trypanosoma Cruzi Tryparedoxin II Interacts with Different Peroxiredoxins under Physiological and Oxidative Stress Conditions. Exp. Parasitol..

[48] González-Chávez Z., Vázquez C., Mejia-Tlachi M., Márquez-Dueñas C., Manning-Cela R., Encalada R., Rodríguez-Enríquez S., Michels P.A.M., Moreno-Sánchez R., Saavedra E. (2019). Gamma-Glutamylcysteine Synthetase and Tryparedoxin 1 Exert High Control on the Antioxidant System in Trypanosoma Cruzi Contributing to Drug Resistance and Infectivity. Redox Biol..

