# Supplementary material for: Overoxidation and Oligomerization of Trypanosoma cruzi Cytosolic and Mitochondrial Peroxiredoxins
Source: Pathogens. 2023 Oct 23;12(10):1273. doi: 10.3390/pathogens12101273 (PMC10610341; doi:10.3390/pathogens12101273)
Supplement: Supplementary file 1 [file pathogens-12-01273-s001.zip › pathogens-2641637-supplementary.pdf]

Supplementary Material; Piñeyro et al. Figure S1.

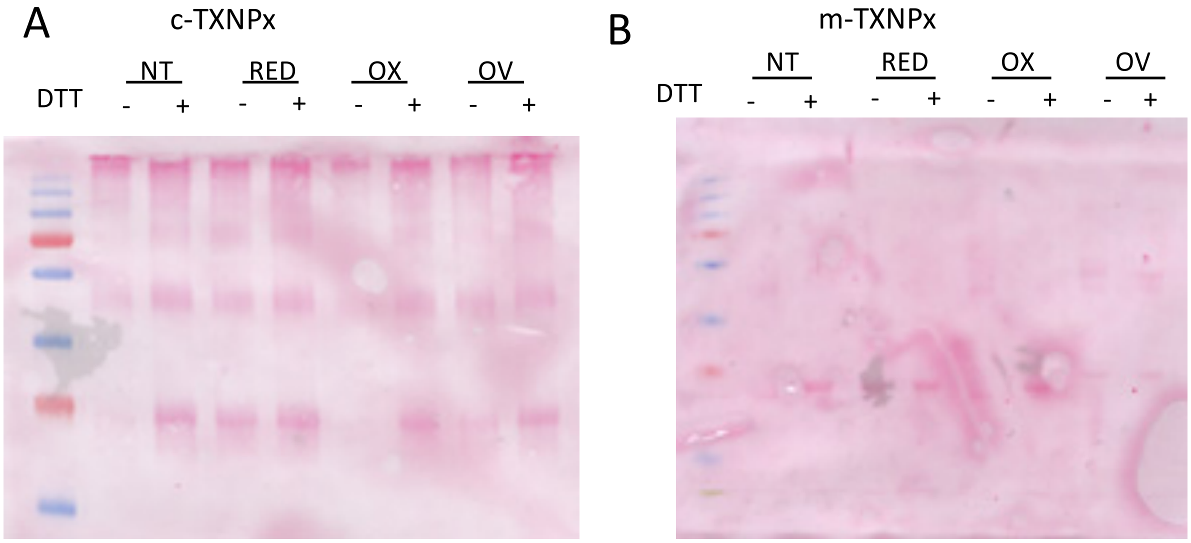

Figure S1. Loading control of western blots shown in Figure 1A.

3 μg of proteins, A: c-TXNPx and B: m-TXNPx, treated: (RED): 10mM DTT; Oxidation (OX): H_2O_2 (1:1 molar ratio); Overoxidation (OV): 10mM DTT and 10mM H_2O_2; NT: proteins without treatment, were resolved by SDS-PAGE, transferred to nitrocellulose membranes and stained with Ponceau-S red as loading control.

Supplementary Material; Piñeyro et al. Figure S2.

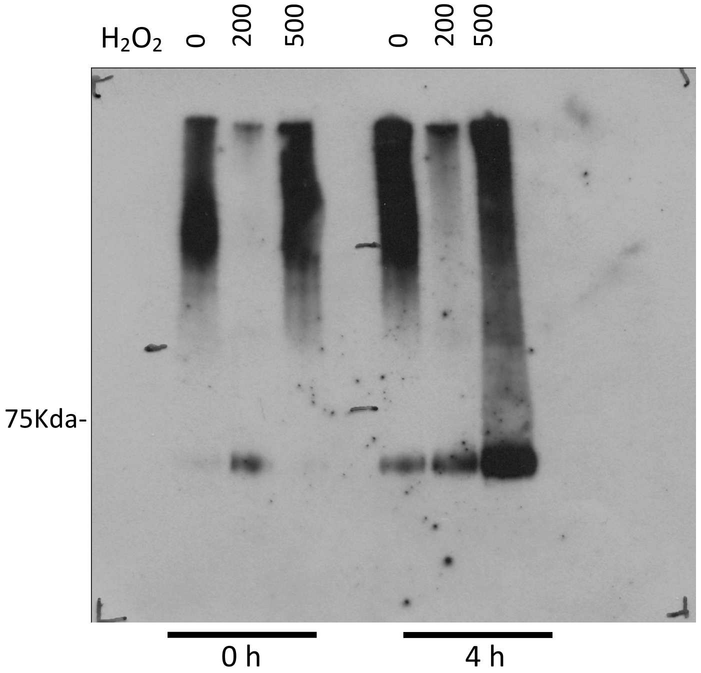

Figure S2. Loading control of western blots shown in Figure 4.

Protein extracts from exponentially growing epimastigotes incubated in PBS-1% glucose at two concentrations of H_2O_2 (200 and 500 μM) and lysed at different times (0 or 4 h) were resolved by Native Page followed by Western blot using anti tubulin antibody (Sigma). The membrane was analyzed with anti-c-TXNPx and anti-m-TXNPx antibodies, stripped and analyzed with anti-tubulin antibody.

Supplementary Material; Piñeyro et al. Figure S3.

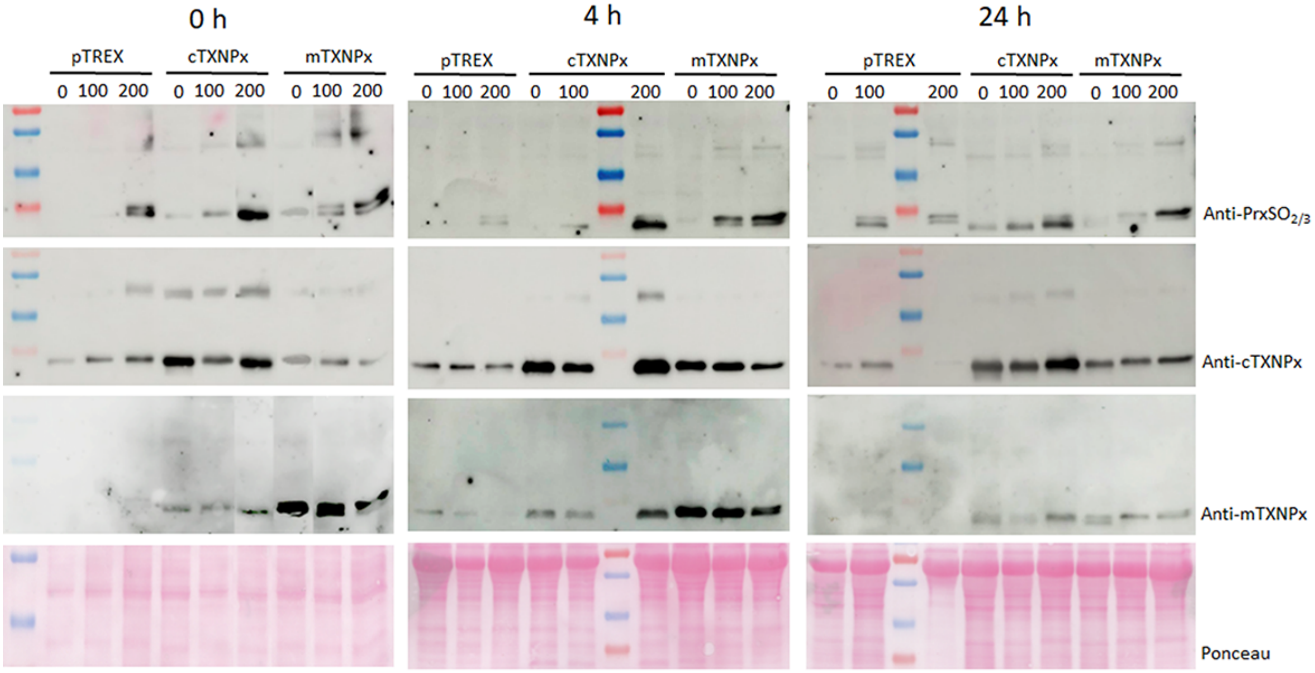

Figure S3. Analysis of the dynamics of overoxidation over time in epimastigotes. Overexpressing c-TXNPx and m-TXNPx lines were treated with 100 and 200 μM H_2O_2 for 30 min in PBS-1% glucose. Parasites were lysed at different times (0, 4, and 24 h) after growth in LIT medium and analyzed by Western Blot with anti- $SO_{2/3}$ (1/1000) and 15 μg of each extract. The membrane was stripped and analyzed for other antibodies: anti-c-TXNPx (1/5000), anti-m-TXNPx (1/1000). The loading control was performed by Ponceau-S red staining of the membrane.

Supplementary Material; Piñeyro et al. Figure S4.

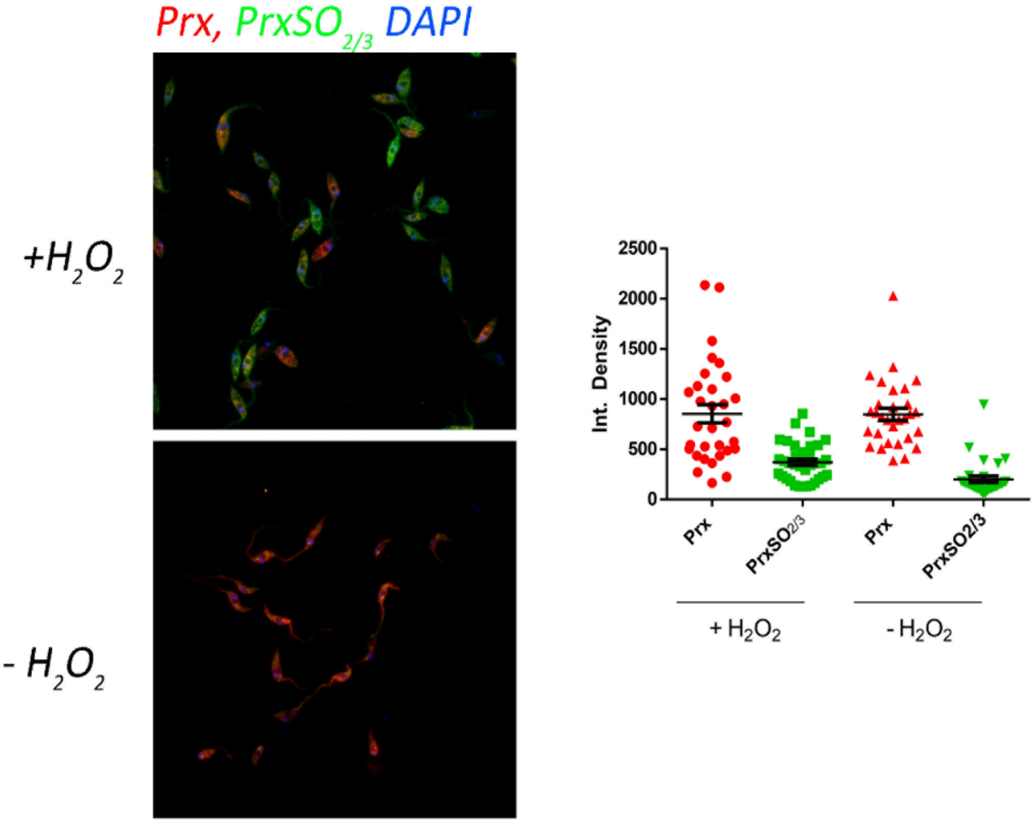

Figure S4. Immunofluorescence of epimastigotes.

Epimastigotes incubated with or without 500 μM H_2O_2 and stained with anti cTXNPx, anti $PrxSO_{2/3}$ or DAPI for nuclear and kinetoplast visualization. The graph shows the integrated density for c-TXNPx fluorescence (red) and $PrxSO_{2/3}$ (green) after incubation with or without 500 μM H_2O_2. Integrated density was calculated with FIJI.

Supplementary Material; Piñeyro et al. Figure S5.

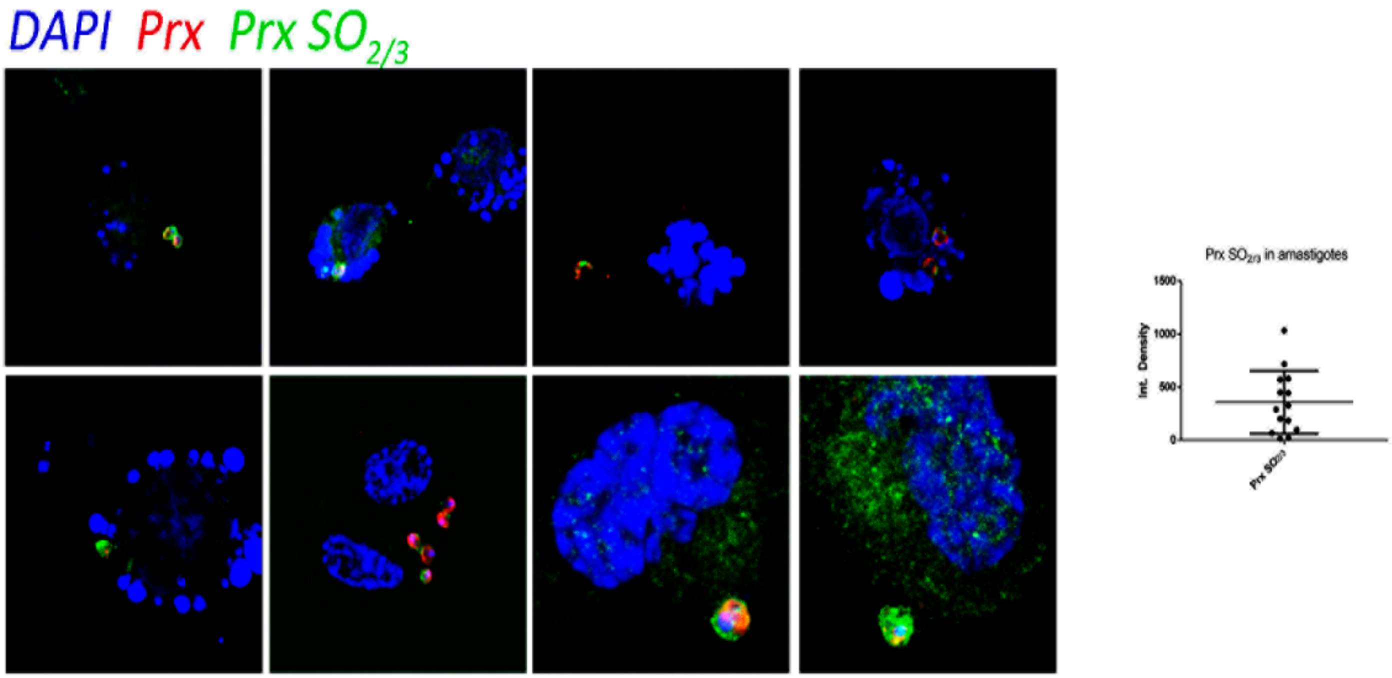

Figure S5. Immunofluorescence of THP1 macrophages infected with trypomastigotes and fixed after internalization.

Cells were stained with DAPI for nuclear and kinetoplast visualization, with anti c-TXNPx and with anti $PrxSO_{2/3}$ antibody. The graph shows the integrated density of $PrxSO_{2/3}$ fluorescence in intracellular amastigotes obtained with FIJI software.

Supplementary Material; Piñeyro et al. Figure S6.

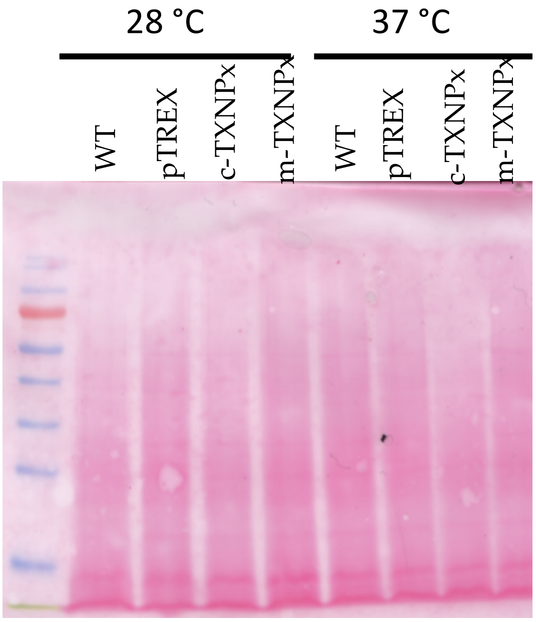

Figure S6. Loading control of western blot shown in Figure 8B.
Proteins extracts (20µl of 3.10[6] cells/mL) of parasites grown at 28°C or 37°C for 72 h, were resolved by SDS-PAGE, transferred to nitrocellulose membranes and stained with Ponceau-S red as loading control.
